# Costing of HIV services, Uganda and United Republic of Tanzania

**DOI:** 10.2471/BLT.22.289580

**Published:** 2023-08-22

**Authors:** Ryan K McBain, Monica Jordan, Ntuli A Kapologwe, Joseph Kagaayi, Elizabeth E Kiracho, Allyala K Nandakumar

**Affiliations:** a Center for Integration Science, Brigham and Women’s Hospital, 75 Francis Street, Boston, MA 02115, United States of America (USA).; b Institute for Global Health and Development, Brandeis University, Waltham, USA.; c President’s Office – Regional Administration, Dar es Salaam, United Republic of Tanzania.; d Department of Health Economics, Makerere University School of Public Health, Kampala, Uganda.

## Abstract

**Objective:**

To evaluate resource allocation and costs associated with delivery of human immunodeficiency virus (HIV) services in Uganda and the United Republic of Tanzania.

**Methods:**

We used time-driven activity-based costing to determine the resources consumed and costs of providing five HIV services in Uganda and the United Republic of Tanzania: antiretroviral therapy (ART); HIV testing and counselling; prevention of mother-to-child transmission; voluntary male medical circumcision; and pre-exposure prophylaxis.

**Findings:**

Country-based teams undertook time-driven activity-based costing with 1119 adults in Uganda and 886 adults in the United Republic of Tanzania. In Uganda, service delivery costs ranged from 8.18 United States dollars (US$) per visit for HIV testing and counselling to US$ 43.43 for ART (for clients in whom HIV was suppressed). In the United Republic of Tanzania, these costs ranged from US$ 3.67 per visit for HIV testing and counselling to US$ 28.00 for voluntary male medical circumcision. In both countries, consumables were the main cost driver, accounting for more than 60% of expenditure. Process maps showed that in both countries, registration, measurement of vital signs, consultation and medication dispensing were the steps that occurred most frequently for ART clients.

**Conclusion:**

Establishing a rigorous, longitudinal system for tracking investments in HIV services that includes thousands of clients and numerous facilities is achievable in different settings with a high HIV burden. Consistent engagement of implementation partners and standardized training and data collection instruments proved essential for the success of these exercises.

## Résumé

**Objectif:**

Analyser l'allocation des ressources et les coûts liés à la fourniture de services de prise en charge du virus de l'immunodéficience humaine (VIH) en Ouganda et en République-Unie de Tanzanie.

**Méthodes:**

Nous avons utilisé un système de calcul des coûts par activité piloté par le temps (TDABC), pour identifier les ressources consommées et les coûts engendrés par cinq services de prise en charge du VIH en Ouganda et en République-Unie de Tanzanie: le traitement antirétroviral (TAR); le dépistage du VIH et les conseils en la matière; la prévention de la transmission de la mère à l'enfant; la pratique volontaire de la circoncision masculine médicalisée; et enfin, la prophylaxie préexposition.

**Résultats:**

Des équipes déployées dans chaque pays ont mené un calcul des coûts par activité piloté par le temps auprès de 1119 adultes en Ouganda et 886 adultes en République-Unie de Tanzanie. En Ouganda, les coûts de prestation des services étaient compris entre 8,18 dollars américains (USD) par visite de dépistage du VIH et de conseils en la matière, et 43,43 USD pour un TAR (sur les patients chez qui la charge virale a été supprimée). En République-Unie de Tanzanie, ces coûts variaient entre 3,67 USD par visite de dépistage du VIH et de conseils en la matière à 28,00 USD pour la pratique volontaire de la circoncision masculine médicalisée. Dans les deux pays, les consommables représentaient le principal facteur de coût, avec plus de 60% des dépenses. Les schémas de procédure ont montré que, dans les deux pays, l'enregistrement, la mesure des signes vitaux, la consultation et l'administration du traitement constituaient les étapes les plus fréquentes chez les patients faisant l'objet d'un TAR.

**Conclusion:**

Il est possible d'établir un dispositif longitudinal rigoureux pour suivre les investissements réalisés dans les services de prise en charge du VIH, qui inclut des milliers de patients et de nombreux établissements dans différents endroits fortement touchés par le VIH. L'implication constante des partenaires de mise en œuvre et la standardisation des formations et instruments de collecte des données se sont révélées essentielles à la réussite de ces démarches.

## Resumen

**Objetivo:**

Analizar la asignación de recursos y los costes asociados a la prestación de servicios por el virus de la inmunodeficiencia humana (VIH) en Uganda y en la República Unida de Tanzania.

**Métodos:**

Utilizamos los costes basados en el tiempo invertido por actividad, con el fin de determinar los recursos consumidos y los costes de la prestación de cinco servicios por VIH en Uganda y en la República Unida de Tanzania: terapia antirretroviral (TAR); pruebas y asesoramiento sobre el VIH; prevención de la transmisión de madre a hijo; circuncisión médica masculina de carácter voluntario y profilaxis previa a la exposición.

**Resultados:**

Los equipos fundados en el país se encargaron de analizar los costes basados en el tiempo invertido por actividad con 1119 adultos en Uganda y 886 adultos en la República Unida de Tanzania. En Uganda, los costes por la prestación de servicios oscilaron entre 8,18 dólares estadounidenses (USD) por visita destinada a pruebas y asesoramiento sobre VIH, y 43,43 USD por TAR (para clientes en los que se había erradicado el VIH). En la República Unida de Tanzania, estos costes estuvieron comprendidos entre 3,67 USD por visita destinada a pruebas y asesoramiento sobre VIH, y 28,00 USD por circuncisión médica masculina de carácter voluntario. En ambos países, los consumibles fueron los principales generadores de costes, representando más del 60% del gasto. Los mapas de procesos mostraron que en ambos países el registro, la medición de las constantes vitales, así como las consultas y la dispensación de medicación fueron los pasos que más se repitieron entre los clientes con TAR.

**Conclusión:**

Implantar un sistema riguroso y longitudinal para realizar un seguimiento de las inversiones en servicios por VIH, lo que engloba a miles de clientes y numerosas instalaciones, es algo que se puede lograr en aquellos centros con una elevada incidencia de VIH. Quedó demostrado que el compromiso constante de los socios encargados de la implementación, así como la existencia de instrumentos estandarizados de formación y de recopilación de datos resultaban esenciales para el éxito de estos ejercicios.

## ملخص

**الغرض:**

تقييم تخصيص الموارد، والتكاليف المرتبطة بتقديم خدمات فيروس نقص المناعة البشرية (HIV) في كل من أوغندا وجمهورية تنزانيا المتحدة.

**الطريقة:**

استخدمنا طريقة لحساب التكاليف على أساس الأنشطة بالاعتماد على الوقت، لتحديد الموارد المستهلكة، وتكاليف توفير خمس خدمات لفيروس نقص المناعة البشرية في أوغندا وجمهورية تنزانيا المتحدة: وهما العلاج المضاد للفيروسات القهقرية (ART)؛ واختبار واستشارات فيروس نقص المناعة البشرية، ومنع انتقال العدوى من الأم إلى الطفل؛ والختان الطبي الطوعي للذكور؛ والعلاج الوقائي قبل التعرض.

**النتائج:**

قامت الفرق في كل دولة بإجراء حساب للتكاليف على أساس الأنشطة بالاعتماد على الوقت مع 1119 شخصًا بالغًا في أوغندا، و886 شخصًا بالغًا في جمهورية تنزانيا المتحدة. في أوغندا، تراوحت تكاليف تقديم الخدمة من 8.18 دولارًا أمريكيًا لكل زيارة لاختبار واستشارات فيروس نقص المناعة البشرية، إلى 43.43 دولارًا أمريكيًا للعلاج المضاد للفيروسات القهقرية (للعملاء الذين تم السيطرة على فيروس نقص المناعة البشرية لديهم). في جمهورية تنزانيا المتحدة، تراوحت هذه التكاليف بين 3.67 دولارًا أمريكيًا لكل زيارة لاختبار واستشارات فيروس نقص المناعة البشرية، إلى 28.00 دولارًا أمريكيًا للختان الطبي الطوعي للذكور. وفي كلتا الدولتين، كانت المواد المستهلكة هي المحرك الرئيسي للتكلفة، حيث شكلت أكثر من %60 من النفقات. وأظهرت خرائط العمليات أن التسجيل، وقياس العلامات الحيوية، والاستشارات وصرف الأدوية، في كلا الدولتين، كانت هي الخطوات الأكثر حدوثًا بالنسبة لعملاء العلاج المضاد للفيروسات القهقرية.

**الاستنتاج:**

إن إنشاء نظام صارم وطولاني لتتبع الاستثمارات في خدمات فيروس نقص المناعة البشرية، التي تشمل آلاف العملاء والعديد من المرافق، هو أمر يمكن تحقيقه في أوضاع مختلفة ذات عبء مرتفع لفيروس نقص المناعة البشرية. وقد أثبتت المشاركة المتسقة لشركاء التنفيذ، وأدوات التدريب الموحدة وجمع البيانات، أنها ضرورية لنجاح هذه الممارسات.

## 摘要

**目的:**

旨在评估乌干达和坦桑尼亚联合共和国提供人类免疫缺陷病毒 (HIV) 服务的相关资源分配和成本。

**方法:**

我们采用了时间驱动的作业成本法，来确定在乌干达和坦桑尼亚联合共和国提供五种 HIV 服务所消耗的资源和成本：抗逆转录病毒治疗 (ART)；HIV 检测和咨询；预防母婴传播；自愿性男性包皮环切术；以及暴露前预防。

**结果:**

基于每个国家的团队对乌干达的 1,119 名成年人和坦桑尼亚联合共和国的 886 名成年人使用时间驱动作业成本法进行了核算。在乌干达，提供服务的费用从每次 HIV 检测和咨询的 8.18 美元到 ART (针对 HIV 受到抑制的患者)的 43.43 美元不等。在坦桑尼亚联合共和国，这些费用从每次 HIV 检测和咨询的 3.67 美元 到自愿性男性包皮环切术的 28 美元不等。在这两个国家，耗材是主要的成本驱动因素，占支出的 60% 以上。流程图显示，在这两个国家，挂号、测量生命体征、咨询和配药是 ART 患者最常进行的步骤。

**结论:**

在 HIV 负担沉重的不同环境中，可以建立一个严格的纵向系统来跟踪对包括数千名患者和众多设施在内的 HIV 服务的投资。事实证明，实施合作伙伴的持续参与以及标准化的培训和数据收集工具对这些活动的成功至关重要。

## Резюме

**Цель:**

Оценить распределение ресурсов и расходы, связанные с оказанием услуг по лечению вируса иммунодефицита человека (ВИЧ) в Объединенной Республике Танзании и Уганде.

**Методы:**

Для определения потребляемых ресурсов и затрат на оказание пяти услуг по борьбе с ВИЧ в Объединенной Республике Танзании и Уганде: антиретровирусной терапии (АРТ), тестирования на ВИЧ и консультирования, профилактики передачи инфекции от матери ребенку, добровольного медицинского обрезания крайней плоти у мужчин и доконтактной профилактики – использовался метод калькуляции расходов за произведенные виды деятельности, основанный на учете времени.

**Результаты:**

Группы, работающие в разных странах, провели калькуляцию расходов за произведенные виды деятельности на основе временной активности с участием 886 взрослых в Объединенной Республике Танзании и 1119 взрослых в Уганде. В Уганде стоимость услуг варьировалась от 8,18 долл. США за визит при тестировании на ВИЧ и консультировании до 43,43 долл. США за АРТ (для клиентов, у которых ВИЧ был подавлен). В Объединенной Республике Танзании эти расходы варьировались от 3,67 долл. США за визит для тестирования на ВИЧ и консультирования до 28,00 долл. США за добровольное медицинское обрезание крайней плоти у мужчин. В обеих странах основным фактором повышения расходов были расходные материалы, на долю которых приходилось более 60% затрат. Технологические карты продемонстрировали, что в обеих странах регистрация, измерение жизненных показателей, консультация и выдача лекарств – это те этапы, которые наиболее часто встречаются у клиентов, получающих АРТ.

**Вывод:**

Создание строгой продольной системы отслеживания инвестиций в оказание услуг по борьбе с ВИЧ, охватывающей тысячи клиентов и множество учреждений, вполне достижимо в различных странах с высокой заболеваемостью ВИЧ. Последовательное участие партнеров по реализации мероприятий, стандартизированные инструменты обучения и сбора данных оказались крайне необходимыми для их успешного проведения.

## Introduction

Despite evolving epidemiological trends, human immunodeficiency virus (HIV) and acquired immunodeficiency syndrome (AIDS) are still responsible for one of the largest disease burdens in sub-Saharan Africa.^1^ In low-income countries such as Uganda and the United Republic of Tanzania, governments contribute about 10% to total spending on HIV and AIDS in their countries, with the remainder provided mostly through development assistance for health.^2^ As these countries move closer to the 90–90–90 targets of the Joint United Nations Programme on HIV/AIDS (UNAIDS),^3^ development assistance for health is evolving; the expectation is that governments will assume incrementally greater financial responsibility for the response to HIV and AIDS over time.^4^ These changes have increased the need to ensure HIV services are efficient, equitable and sustainable.^5^

Nevertheless, how financial and human resources are allocated at health facilities throughout each country are still unclear. Historically, Uganda and the United Republic of Tanzania have implemented a variety of activities to understand HIV-related costs in regions of each country.^6^^,^^7^ A long-term strategy for measuring allocation of resources would be to institute an ongoing national performance management system: one that tracks expenditure and provision of care across clients, facilities and geographic regions of each country.^8^

The activity-based costing and management initiative, established in 2020, aims to achieve this objective, and by extension to support alignment and optimization of investments in the HIV response between governments and international partners.^9^ This initiative is currently being introduced in five African countries (Kenya, Mozambique, Namibia, Uganda and United Republic of Tanzania), with Uganda and the United Republic of Tanzania being the earliest implementers. Lead partner institutions include country health ministries and administrations, the Office of the United States Global AIDS Coordinator and Health Diplomacy, and the United States Agency for International Development (USAID). Other stakeholders include the Global Fund to Fight AIDS, Tuberculosis and Malaria, UNAIDS; the US Centers for Disease Control and Prevention; and the US Department of the Treasury.

Central to activity-based costing and management is the application of time-driven activity-based costing, a cost accounting approach that involves real-time observation of clients receiving services across a wide range of facilities. Time-driven activity-based costing therefore shows the costs of the actual allocation of resources – typically measured in minutes consumed – for each client. This information permits researchers to measure variation in the allocation of resources from client to client and facility to facility. Similarly, by aggregating observations across clients, data collectors can inductively model the actual care pathways for service delivery, often described as process maps. They can then compare these process maps to guidelines or best practices to assess quality of care.

In this study, we report on the implementation, results and impact of the use of activity-based costing and management in Uganda and the United Republic of Tanzania. We focus on three main sets of observations: refinement of cost estimates and cost drivers; construction of process maps to assess existing care pathways; and establishment of a data visualization system for assessing key performance indicators. As other countries are considering using this method to optimize HIV services at regional and national levels, we also highlight lessons learned from the two examples of activity-based costing and management. 

## Methods

### Setting

Uganda and the United Republic of Tanzania are low-income countries with a high HIV burden. Gross domestic product (GDP) is close to 1000 United States dollars (US$) per capita in each country^10^^,^^11^ and HIV prevalence in adults is about 5%.^12^^,^^13^ Both countries have rapidly growing economies and disease burdens. Before the coronavirus disease 2019 (COVID-19) pandemic, annualized GDP growth was 6% in both countries.^14^^,^^15^ Meanwhile, the estimated proportion of deaths caused by noncommunicable diseases grew from about 25% in 2010 to 35% in 2020 in both countries.^1^

These trends prompted each country to channel greater resources into health care^16^^–^^18^ and to adjust HIV services. For example, in 2016, the United Republic of Tanzania established different service delivery models for HIV services, including for antiretroviral therapy (ART), whereby clients in whom the virus is suppressed receive streamlined and less frequent ART visits.^19^ In 2017, Uganda did the same and introduced a five-tier model for ART.^20^ While such guidelines should, in theory, influence resource-allocation decisions and costs, the practical outcome is still unclear.^7^^,^^20^

Both countries launched activity-based costing and management in early 2020, based on the interest of health ministry officials to quantify HIV resource allocation and costs. Data collection in the United Republic of Tanzania took place in late 2020, while data collection in Uganda took place between late 2020 and early 2021. Table 1 gives an overview of the composition of facilities at which data were collected, by country and region. The sampling frame in both countries was the national register of HIV facilities, from which study clinics were purposively selected based on region, urban–rural distribution, public versus private ownership, level of support by the US President’s Emergency Plan for AIDS Relief (PEPFAR), and HIV service volume.

**Table 1:** Characteristics of facilities included in costing of HIV services, Uganda and United Republic of Tanzania, 2020–2021

Type of location and region	No. (%)	Urban/rural, no. (%)	Public/private, no. (%)	Supported by PEPFAR, no. (%)		HIV client volume, no. (%)^a^		
						Low	Medium	High
**Uganda^b^**								
**Central**	8 (100)	5 (62) / 3 (38)	5 (62) / 3 (38)	8 (100)		0 (0)	5 (62)	3 (38)
Health centre II	1 (12)	0 (0) / 1 (100)	0 (0) / 1 (100)	1 (100)		0 (0)	1 (100)	0 (0)
Health centre III	4 (50)	1 (25) / 3 (75)	3 (75) / 1 (25)	4 (100)		0 (0)	3 (75)	1 (25)
Health centre IV	2 (25)	1 (50) / 1 (50)	2 (100) / 0 (0)	2 (100)		0 (0)	1 (50)	1 (50)
Hospital	1 (12)	1 (100) / 0 (0)	0 (0) / 1 (100)	1 (100)		0 (0)	0 (0)	1 (100)
**Eastern**	8 (100)	5 (62) / 3 (38)	8 (100) / 0 (0)	8 (100)		1 (12)	6 (75)	1 (12)
Health centre II	0 (0)	NA	NA	NA		NA	NA	NA
Health centre III	5 (62)	2 (40) / 3 (60)	5 (100) / 0 (0)	5 (100)		1 (20)	4 (80)	0 (0)
Health centre IV	2 (25)	1 (50) / 1 (50)	2 (100) / 0 (0)	2 (100)		0 (0)	2 (100)	0 (0)
Hospital	1 (12)	0 (0) / 1 (100)	1 (100) / 0 (0)	1 (100)		0 (0)	0 (0)	1 (100)
**Northern**	8 (100)	5 (62) / 3 (38)	5 (62) / 3 (38)	8 (100)		1 (12)	4 (50)	3 (38)
Health centre II	0 (0)	NA	NA	NA		NA	NA	NA
Health centre III	4 (50)	1 (25) / 3 (75)	3 (75) / 1 (25)	4 (100)		1 (25)	3 (75)	0 (0)
Health centre IV	3 (38)	2 (67) / 1 (33)	2 (67) / 1 (33)	3 (100)		0 (0)	0 (0)	3 (100)
Hospital	1 (12)	0 (0) / 1 (100)	0 (0) / 1 (100)	1 (100)		0 (0)	1 (100)	0 (0)
**Western**	7 (100)	7 (100) / 0 (0)	6 (86) / 1 (14)	7 (100)		0 (0)	1 (14)	6 (86)
Health centre II	0 (0)	NA	NA	NA		NA	NA	NA
Health centre III	2 (28)	0 (0) / 2 (100)	2 (100) / 0 (0)	2 (100)		0 (0)	1 (50)	1 (50)
Health centre IV	3 (43)	0 (0) / 3 (100)	3 (100) / 0 (0)	3 (100)		0 (0)	0 (0)	3 (100)
Hospital	2 (28)	0 (0) / 2 (100)	1 (50) / 1 (50)	2 (100)		0 (0)	0 (0)	2 (100)
**United Republic of Tanzania**								
**Dar Es Salaam**	3 (100)	3 (100) / 0 (0)	3 (100) / 0 (0)	3 (100)		1 (33)	0 (0)	2 (67)
Dispensary	1 (33)	1 (100) / 0 (0)	1 (100) / 0 (0)	1 (100)		1(100)	0 (0)	0 (0)
Health centre	1 (33)	1 (100) / 0 (0)	1 (100) / 0 (0)	1 (100)		0 (0)	0 (0)	1(100)
Hospital	1 (33)	1 (100) / 0 (0)	1 (100) / 0 (0)	1 (100)		0 (0)	0 (0)	1(100)
**Dodoma**	3 (100)	1 (33) / 2 (67)	2 (67) / 1 (33)^c^	3 (100)		0 (0)	2 (67)	1 (33)
Dispensary	0 (0)	NA	NA	NA		NA	NA	NA
Health centre	2 (67)	1 (50) / 1 (50)	2 (100) / 0 (0)	2 (100)		0 (0)	1 (50)	1 (50)
Hospital	1 (33)	0 (0) / 1 (100)	0 (0) / 1 (100)	1 (100)		0 (0)	1 (100)	0 (0)
**Kagera**	2 (100)	0 (0) / 2 (100)	2 (100) / 0 (0)	2 (100)		0 (0)	0 (0)	2 (100)
Dispensary	0 (0)	NA	NA	NA		NA	NA	NA
Health centre	1 (50)	0 (0) / 1 (100)	1 (100) / 0 (0)	1 (100)		0 (0)	0 (0)	1 (100)
Hospital	1 (50)	0 (0) / 1 (100)	1 (100) / 0 (0)	1 (100)		0 (0)	0 (0)	1 (100)
**Mbeya**	4 (100)	1 (25) / 3 (75)	4 (100) / 0 (0)	4 (100)		0 (0)	2 (50)	2 (50)
Dispensary	0 (0)	NA	NA	NA		NA	NA	NA
Health centre	3 (75)	1 (33) / 2 (67)	3 (100) / 0 (0)	3 (100)		0 (0)	2 (67)	1 (33)
Hospital	1 (25)	0 (0) / 1 (100)	1 (100) / 0 (0)	1 (100)		0 (0)	0 (0)	1 (100)
**Mwanza**	3 (100)	1 (33) / 2 (67)	3 (100) / 0 (0)	3 (100)		1 (33)	1 (33)	1 (33)
Dispensary	2 (67)	1 (50) / 1 (50)	2 (100) / 0 (0)	2 (100)		1 (50)	1 (50)	0 (0)
Health centre	0 (0)	NA	NA	NA		NA	NA	NA
Hospital	1 (33)	0 (0) / 1 (100)	1 (100) / 0 (0)	1 (100)		0 (0)	0 (0)	1 (100)
**Njombe**	3 (100)	1 (33) / 2 (67)	2 (67) / 1 (33)^b^	30 (100)		0 (0)	1 (33)	2 (67)
Dispensary	0 (0)	NA	NA	NA		NA	NA	NA
Health centre	2 (67)	1 (50) / 1 (50)	2 (100) / 0 (0)	2 (100)		0 (0)	1 (50)	1 (50)
Hospital	1 (33)	0 (0) / 1 (100)	0 (0) / 1 (100)	1 (100)		0 (0)	0 (0)	1 (100)
**Tabora**	4 (100)	0 (0) / 4 (100)	3 (75) / 1 (25)	3 (75)		2 (50)	1 (25)	1 (25)
Dispensary	2 (50)	0 (0) / 2 (100)	1 (50) / 1 (50)	1 (50)		1 (50)	1 (50)	0 (0)
Health centre	1 (25)	0 (0) / 1 (100)	1 (100) / 0 (0)	1 (100)		1(100)	0 (0)	0 (0)
Hospital	1 (25)	0 (0) / 1 (100)	1 (100) / 0 (0)	1 (100)		0 (0)	0 (0)	1 (100)

HIV: human immunodeficiency virus; NA: not applicable; PEPFAR: United States President’s Emergency Plan for AIDS Relief.

^a^ The definition of HIV client volume is: low (20–249 clients with HIV a month); medium (250–1249 clients a month); high (≥ 1250 clients a month).

^b^ Faith-based organization.

^c^ In Uganda, health centre II facilities serve a few thousand people (parish level), provide screening and treatment for common health conditions, and have about five staff. Health centre III facilities serve several thousand people, provide more comprehensive screening and treatment, and have about 20 staff. Health centre IV facilities operate at the county level, offer a wide range of outpatient and acute care services, and generally have more than 20 staff.

### Study design

The study used a cross-sectional, observational design. The data collection team gave a half-day presentation to the staff at HIV facilities, which explained the reasons for data collection and the procedures that would be performed by the team. The team followed clients during a 2–4-week period at each facility.

We defined the scope of activity-based costing and management operations in consultation with in-country steering committees. These committees comprised members from the health ministry, office of the president, regional and local administrations, countrywide commissions concerned with HIV and AIDS, and bureaus of statistics, finance and planning. Representatives of international partner organizations mentioned in the introduction also participated. The objectives of the steering committee were to: provide guidance on logistics; ensure that operations answered the questions prioritized by the government; validate data; and promote dissemination of findings. Committee members received bimonthly progress briefings throughout the data collection and analysis period.

We issued requests for proposals to identify local implementation partners who would lead the data collection and analysis. We evaluated responses to these requests based on institutional competencies, previous experience, team composition, and ability to perform the work within a predetermined budget and timeframe. The institutions selected were Makerere University in Uganda and Muhimbili University of Health and Allied Sciences in the United Republic of Tanzania. Over a 2-week period, experts from the USA trained members of these institutions in three areas: (i) the time-driven activity-based costing method and corresponding data collection forms; (ii) implementation protocols, including those related to COVID-19 restrictions; and (iii) electronic tablet use and data storage. The training used a standard curriculum to help ensure cross-country comparability of inputs and cost estimates.

### Participants

Clients were eligible to participate if they met three inclusion criteria: age 18 years or older; attending a participating facility during the data collection period; and seeking one of the five HIV services of interest – ART, HIV testing and counselling, prevention of mother-to-child transmission (PMTCT), voluntary male medical circumcision and pre-exposure prophylaxis. We categorized clients receiving ART as ART-stable, that is clients in whom the virus was suppressed; and ART-unstable, that is clients in whom the virus was not suppressed.

The team screened participants for eligibility during registration at facilities, except for voluntary male medical circumcision services, which were mainly offered through outreach efforts. Team members gave participants a written or verbal consent form, depending on their level of reading comprehension, and assigned them a unique identifier. The following local and international institutional review boards approved the study protocols: National Institute for Medical Research, United Republic of Tanzania; Uganda National Council for Science and Technology; and HML IRB, USA.

### Procedures

Implementation of the time-driven activity-based costing followed six steps, aligned with best practices (Box 1).^21^

Box 1 **Steps in time-driven activity-based costing**

***1. Select medical services and populations***

The scope of our analysis was limited to the inclusion criteria outlined in the procedures section.

***2. Define the care delivery value chain***

Data collectors recorded all potential activities and resources that clients might consume, based on key informant interviews, which were 30-minute, structured in-person interviews with facility leaders, and adapted the content of data collection forms accordingly.

***3. Obtain time estimates for activities***

We gave data collectors stop watches and trained them to observe clients at each step in the care delivery process. They documented the location, length of the client–provider interaction, who was involved, equipment used and consumables allocated to the client over the course of care.

***4. Gather unit cost information***

We obtained cost estimates of supplying client care from facility ledgers, electronic systems and price lists. We included the costs of support staff and overhead expenses as indirect costs.

***5. Estimate the practical capacity of resources***

We gathered information through key informant interviews on how many minutes per year each resource was available to a client at the facility. We calculated the capacity cost rates by dividing the total cost of each resource by its practical capacity, that is, the number of minutes available for use of the resource by clients over a fixed period (1 year).

***6. Calculate the total cost of client care***

We calculated client costs by multiplying capacity cost rates by the number of minutes each client used resources, then multiplying this cost by the quantity of consumables and aggregating across all steps of the care delivery. We estimated annual costs for antiretroviral therapy, and considered all other services one-time encounters.

To ensure conformity of procedures, we applied three measures. First, all data collectors participated in a 1-week training course with instruction and role-playing run by Health Policy Plus (an interorganizational entity based in the USA), after which they needed to pass a final examination. Second, we provided data collectors with a standardized set of data collection instruments. Third, supervisors reviewed data on a routine basis each week to provide feedback to the data collection team.

Data collection itself had four categories: direct observation of clients; client interviews, through a brief exit survey; interviews with providers and staff to gather information on their schedules, annual leave and compensation; and gathering of facility-based information on unit costs for equipment and consumables.

We obtained unit costs from four sources: invoices, receipts, catalogued expenses and payroll information stored in financial management systems. We valued staff time at full salary cost, inclusive of allowances for vacation and benefits. We annualized capital costs for equipment and infrastructure over the useful life of the item, assuming linear depreciation. We assigned unit costs for 2020 to one of five cost categories: human resources, clinic space, equipment, consumables and indirect costs (for example, utilities and overheads). We adjusted expenditure that occurred before 2020 for inflation.

After completing data gathering, the data collection team generated process maps for each HIV service at each facility. Process maps are visual representations of care pathways, showing the where, what, when, who and how often of service delivery. The maps were generated using mean values for continuous measures (for example, average duration of a consultation), and modal averages for discrete measures (for example, most frequent provider performing a consultation).

### Analyses

From a computational perspective, time-driven activity-based costing reduces costs to a single, standardized unit: capacity cost rates. Capacity cost rates are the cost of a resource, such as a provider, physical space or unit of equipment, per minute of available capacity. For example, if a nurse had 100 000 minutes available a year to provide clinical care and received an annual salary of US$ 20 000, the capacity cost rate of this nurse would be US$ 20 000/100 000 minutes = US$ 0.20 per minute. Thus, if a nurse performed an activity such as measurement of vital signs for 5 minutes, the cost of the nurse’s labour would be estimated as: US$ 0.20 per minute × 5 minutes = US$ 1.00.

We estimated the cost per client per visit for ART, HIV testing and counselling, PMTCT, voluntary male medical circumcision and pre-exposure prophylaxis. We also generated aggregate estimates by calculating the median cost per client per service at each facility and overall across facilities within a country. We measured cost drivers according to five cost categories: human resources (labour); physical space (infrastructure); equipment; consumables including medicines; and indirect costs. We used Stata version 17.0 (StataCorp. LP, College Station, USA) for all calculations.

We separated the process maps by service line and the characteristics on which facilities were originally selected, for example, urban versus rural. We used Lucidchart (Lucid Software Inc., South Jordan, USA) to construct process maps.

## Results

### Clients

Data collectors covered 31 facilities in Uganda and 22 in the United Republic of Tanzania (53 facilities in total), and directly observed 1119 adult clients in Uganda and 886 clients in the United Republic of Tanzania. In Uganda, 298 (27%) clients attended clinics for HIV testing and counselling; 285 (25%) for PMTCT; 273 (24%) for ART-unstable; 233 (21%) for ART-stable; 19 (2%) for voluntary male medical circumcision; and 17 (2%) for pre-exposure prophylaxis. In the United Republic of Tanzania, 240 (27%) clients attended clinics for ART-stable; 214 (24%) for ART-unstable; 200 (23%) for PMTCT; 198 (22%) for HIV testing and counselling; 19 (2%) for voluntary male medical circumcision; and 15 (2%) for pre-exposure prophylaxis. 

### Costs and cost drivers

**Fig. 1:**
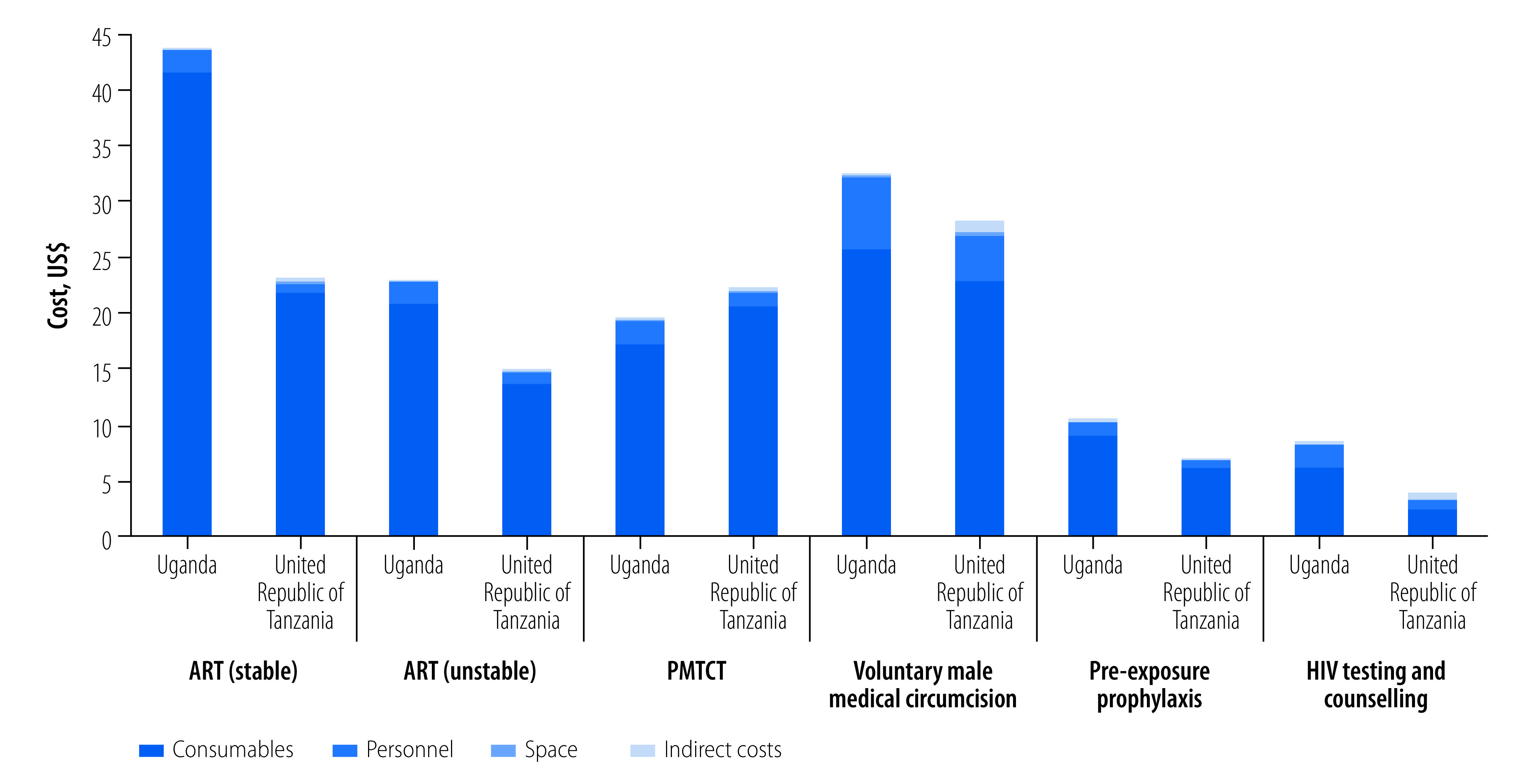
Service delivery costs of HIV services provided, Uganda and United Republic of Tanzania, 2020–2021

ART: antiretroviral therapy; HIV: human immunodeficiency virus; PMTCT: prevention of mother-to-child transmission; US$: United States dollars..

In Uganda, the cost per client per visit ranged from US$ 8.18 for HIV testing and counselling to US$ 43.43 for ART-stable (Fig. 1). This cost in the United Republic of Tanzania ranged from US$ 3.67 for HIV testing and counselling to US$ 28.00 for voluntary male medical circumcision (Fig. 1). The main cost driver in both settings was consumables, ranging from 61% (US$ 2.24/3.67) to 95% (US$ 21.49/22.72) of costs for both countries. By comparison, human resources represented 3% (US$ 0.75/22.72) to 22% (US$ 0.82/3.67) of costs; indirect items represented 1% (US$ 0.16/19.34) to 14% (US$ 0.50/3.67) of costs; and space and equipment collectively accounted for < 1% (US$ 0.06/32.28) to 3% (0.11/3.67) of costs.

### Process maps

**Fig. 2:**
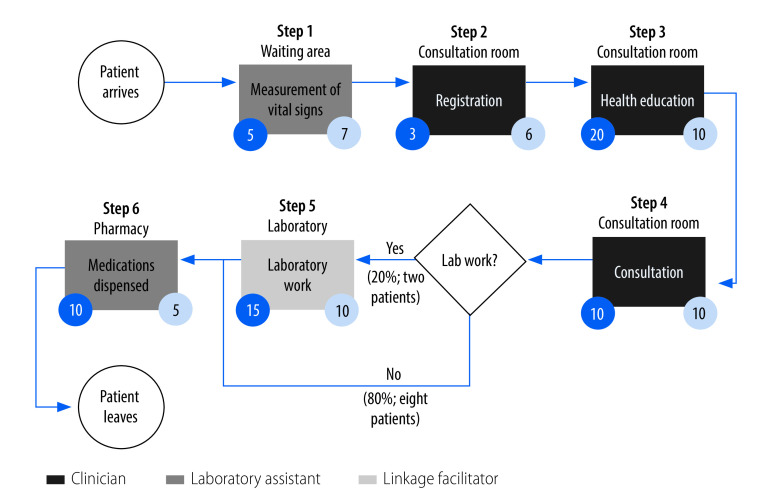
Steps in delivery of care for clients with stable HIV, Amach health centre IV, Uganda, 2020–2021

**Fig. 3:**
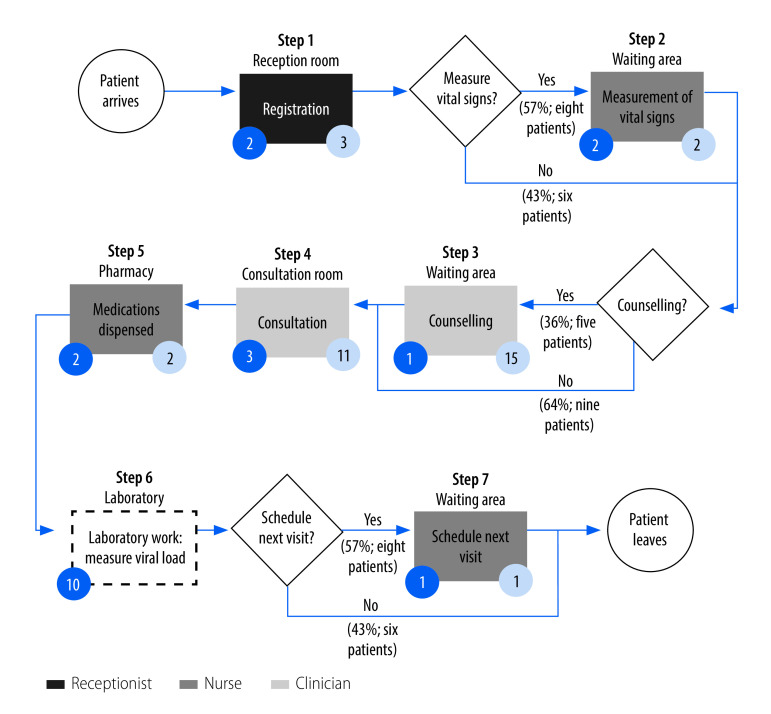
Steps in delivery of care for clients with stable HIV, Njombe health centre, United Republic of Tanzania, 2020

Fig. 2 and Fig. 3 show examples of process maps for delivery of ART services at health centres in Uganda and the United Republic of Tanzania, respectively. 

HIV: human immunodeficiency virus.

Notes: Amach is a medium-sized health centre in northern Uganda. Circles represent entry and exit points from the health system. Rectangles represent steps in the delivery of care. Diamonds represent decisions. Small left-hand circles in dark blue represent the expected duration of the activity (in minutes) and small right-hand circles in light blue represent the observed duration. The figures in brackets are the percentages of clients receiving or not receiving the step. A linkage facilitator is a type of lay health worker in Uganda.

HIV: human immunodeficiency virus.

Notes: Circles represent entry and exit points from the health system. Rectangles represent steps in the delivery of care. Diamonds represent decisions. Small left-hand circles in dark blue represent the expected duration of the activity (in minutes) and small right-hand circles in light blue represent the observed duration. The uncoloured rectangle with a dashed outline represents a step that was skipped. The figures in brackets are the percentages of clients receiving or not receiving the step.

The steps that occurred most of the time for clients receiving ART at Amach health centre IV in northern Uganda were registration, measurement of vital signs, health education, consultation and dispensing of medication (Fig. 2). The only step that was routinely skipped by clients was diagnostics; only 20% (2/10) of clients had laboratory work done. Clinicians performed the registration, health education and consultations, while a linkage facilitator (lay health worker) measured vital signs and dispensed medications, and a laboratory assistant performed diagnostic work. We also observed variation in anticipated versus observed duration of activities: for example, key informants anticipated health education sessions would last 20 minutes, but we observed a health education session was typically just 10 minutes.

Routine steps for clients receiving ART at Njombe Health Centre in the United Republic of Tanzania were registration, measurement of vital signs, consultation, dispensing of medications and scheduling of the next appointment (Fig. 3). Counselling was frequently skipped with only 36% (5/14) of clients receiving this service. Similarly, laboratory work (viral load measurement) was not observed. Regarding the provider of the service, most of the time a nurse measured vital signs, dispensed medication and scheduled the next appointment, while a clinician undertook counselling and consultations, and a receptionist managed the registration. We also observed that the amount of time anticipated for specific activities differed for the observed time: for example, key informants anticipated consultations would last 3 minutes, but the median duration was 11 minutes.

### Key performance indicators

**Fig. 4:**
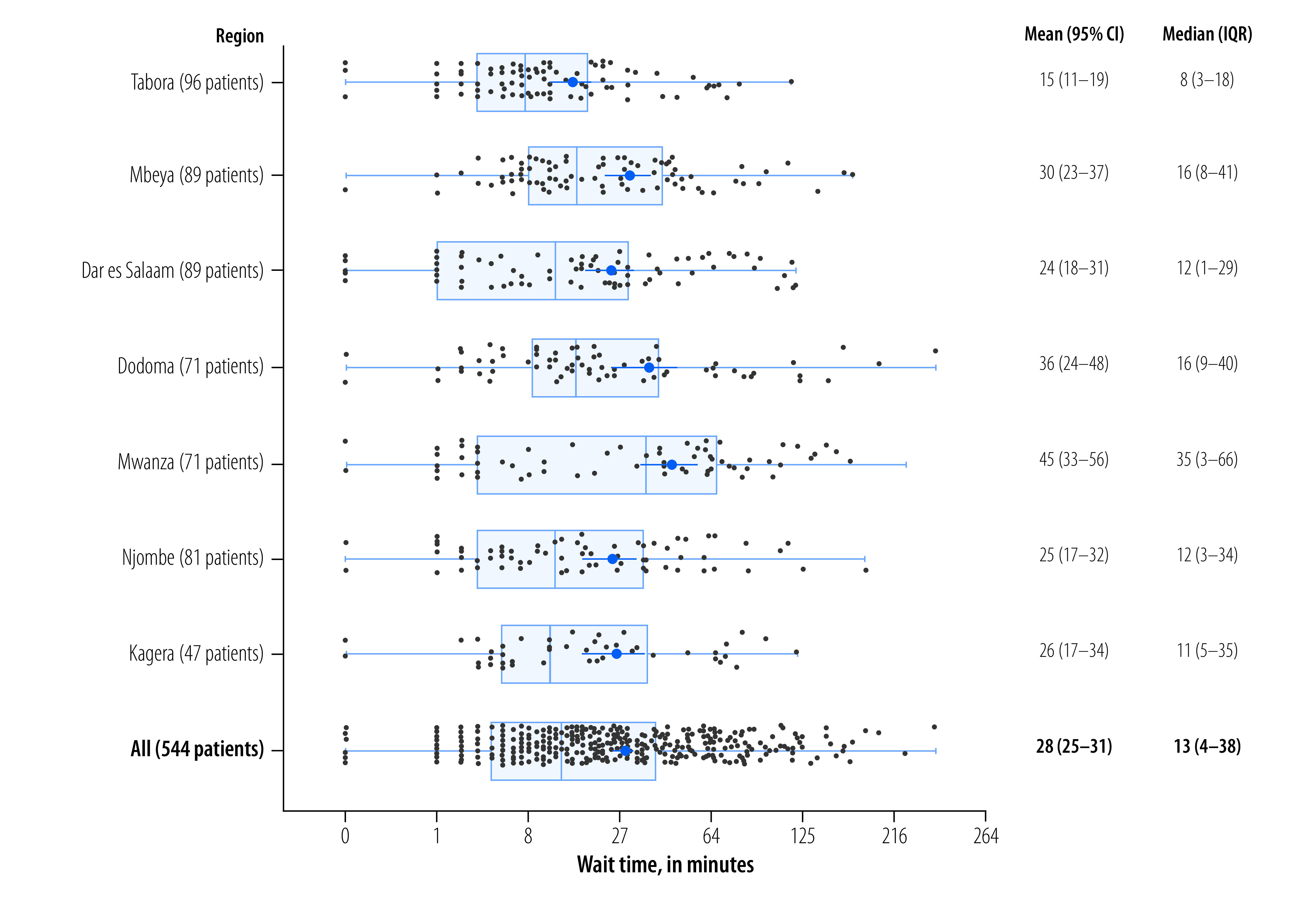
Wait times for services for antiretroviral therapy, by region, United Republic of Tanzania, 2020

CI: confidence interval; IQR: interquartile range.

Notes: Boxplot whiskers show minimum and maximum values; the lower value of the box represents the 25th centile, the mid-line represents the 50th centile and the upper value represents the 75th centile. Blue circles represent mean values and blue whiskers represent 95% CI of the mean. Each black dot represents a client directly observed through time-driven activity-based costing, as well as their corresponding wait time in minutes.

We developed an online key performance indicator dashboard for clinic and government officials to explore activity-based costing and management data.^22^^,^^23^ In the main page of the dashboard, users can select key performance indicators of interest as well as grouping variables such as region or facility. Scatterplots then display the value of each individual client, according to the key performance indicator and grouping variables. For example, Fig. 4 shows the average wait time for clients receiving ART in the United Republic of Tanzania, by region of the country, which indicates that mean wait time in Tabora was 15 minutes compared with 45 minutes in Mwanza.

**Fig. 5:**
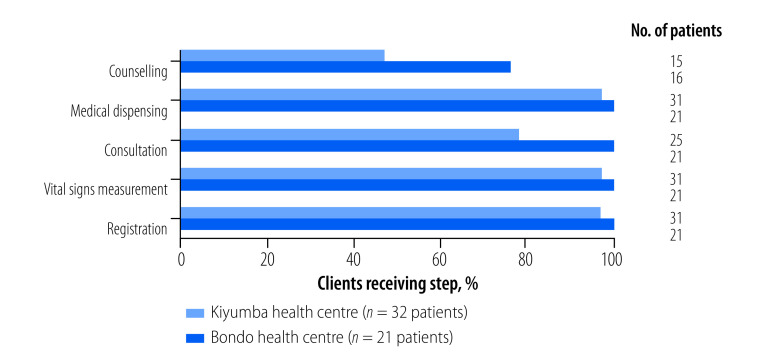
Percentage of clients receiving each step in service delivery of antiretroviral therapy at two health centres, Uganda, 2020

Note: Bondo is a small health centre in Uganda, while Kiyumba is a medium-sized health centre.

**Fig. 6:**
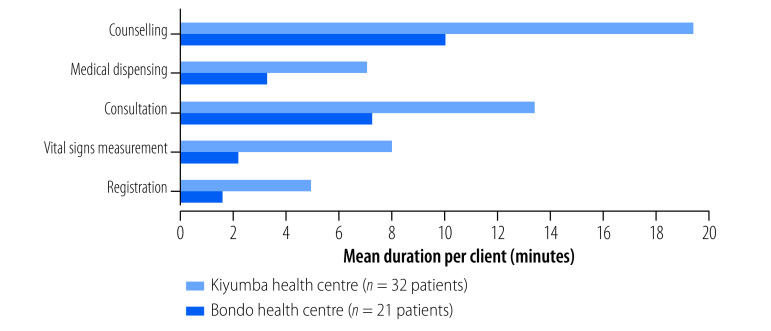
Mean duration of each step in service delivery of antiretroviral therapy at two health centres, Uganda, 2020

**Fig. 7:**
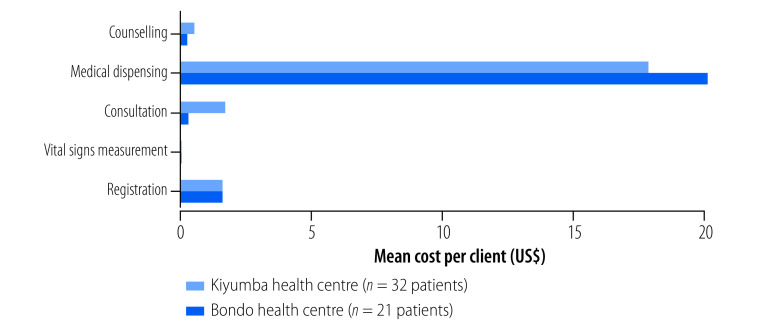
Mean cost of each step in service delivery of antiretroviral therapy at two health centres, Uganda, 2020

HIV: human immunodeficiency virus; US$: United States dollars.

Note: Bondo is a small health centre receiving 20–249 HIV patients per month, while Kiyumba is a medium-sized health centre, receiving 250–1249 patients per month.

Other tabs of the dashboard system allow users to examine additional comparison points, including: a side-by-side comparison of the care delivery processes at two selected facilities; a breakdown of costs and cost drivers for individual interventions at a selected facility; and detailed personnel cost information, including variation in capacity cost rates. Comparisons of the frequency, duration and costs of steps in provision of ART at two health centres in Uganda are shown in Fig. 5, Fig. 6 and Fig. 7. The figures show consultations and counselling occurred more frequently at Bondo health centre, but time interaction between providers and clients was greater at Kiyumba health centre. On balance, the costs were similar.

HIV: human immunodeficiency virus.

Note: Bondo is a small health centre, receiving 20–249 HIV patients per month, while Kiyumba is a medium-sized health centre, receiving 250–1249 patients per month.

## Discussion

We found that activity-based costing and management can be implemented on a wide scale using a rigorous approach to cost accounting if certain elements are in place, such as: strong implementation partners; data collectors who are trained over a 1-week period to use standardized data collection forms; significant supportive supervision; and ongoing oversight from steering committees. Given that the activity-based costing and management operations in Uganda and the United Republic of Tanzania are baseline efforts, we expect the full potential of this operation will be realized in the next 2–3 years. In the interim, these data offer several key insights.

First, consistent with the literature,^24^^–^^26^ we observed that the costs of HIV services were largely driven by consumables. This finding underscores the importance of a stable supply chain, secure storage and distribution mechanisms, and ongoing subsidies to ensure affordability for clients.^27^ Human resources, the second-largest cost driver, varied considerably in terms of tasks and time commitments, from one facility to the next. This finding provides a starting point for discussing standardization and best practices at different levels of care within the countries’ health systems. Second, process maps shed light on the composition of existing care delivery pathways, including departures from expected procedures. Both facility administrators (for example, chief medical officers) and government officials have indicated the importance of documenting such departures to inform initiatives to improve clinical quality.^28^ However, the maps shown in our study are only illustrative. A comprehensive overview of the process maps generated in our study is given in a previous report.^29^ Third, health ministries and government agencies were enthusiastic about using the online key performance indicator system to gather and share information on variations in service delivery from facility to facility and region to region.

Two main challenges were noted with activity-based costing and management: direct observation of patients is onerous, and it can be difficult to ensure patient privacy during consultation. To manage the first issue, time schedules were extended at each facility to allow adequate time for data collection. To protect patient privacy, data collection teams also devised work-around systems: for example, remaining outside consultation rooms until the end of the consultation and then speaking with the provider to gather relevant information (for example, use of equipment).

On a conceptual level, the comprehensive scope of activity-based costing and management operations risked disengagement of facility administrators in the dissemination phase of this work, as individual facilities were only a small part of the overall exercise. To counteract this problem, implementation partners held follow-up conversations with the leadership of individual facilities, in which process maps, cost estimates and other clinical information were shared. Furthermore, as part of a broader learning collaborative, the activity-based costing and management initiative has held regional meetings on a roughly 6-monthly basis, which brought together stakeholders from different countries to share lessons learned and applications of this work. These dissemination efforts have helped ensure sustainability of the initiative. Similarly, ministries of health and finance have considered routine intervals in which to re-do time-driven activity-based costing, using the same set of training and data collection materials to ensure consistency of quality and information over time.

As noted earlier, the next 2–3 years will be a turning point for activity-based costing and management, over which progress and re-aligned investments will be charted and discussed. Equally, implementation of time-driven activity-based costing on a routine basis will require its integration into health management information systems such as DHIS2. Alternatively, countries will need to establish a new information system structure specific to this effort.

We note four limitations of our study, which governments that are thinking of participating in the activity-based costing and management initiative may consider. First, while time-driven activity-based costing can identify inefficiencies within health systems – such as client wait time or provider idle time – it does not offer a strategy to solve these problems. Rather, it provides relevant background for considering reforms within the health system. Second, the activity-based costing and management initiative does not link investments to client outcomes, except by proxy. For example, activity-based costing and management observes the proportion of clients who receive counselling, medication and laboratory services, each of which may be considered an important component of a particular service. However, linking these inputs to outcomes such as client morbidity and mortality would require more robust longitudinal monitoring systems. Third, some aspects of data gathering for time-driven activity-based costing, such as work hours of personnel, were self-reported and therefore may be influenced by social desirability bias. Lastly, we did not estimate the cost of implementing time-driven activity-based costing operations, which could be relevant for countries considering the financial feasibility of participating in this initiative.

To conclude, the activity-based costing and management initiative has provided new information for countries. This information will allow governments to examine their investments in combatting HIV by observing the actual consumption of resources by individual clients, across hundreds of clients and dozens of facilities. The success of this initiative to date reflects a strong commitment by country governments and partner institutions to ensure high-quality care for all people living with HIV.
